# Exosomal miRNAs species in the blood of small cell and non-small cell lung cancer patients

**DOI:** 10.18632/oncotarget.24857

**Published:** 2018-04-13

**Authors:** Valeriy Poroyko, Tamara Mirzapoiazova, Arin Nam, Isa Mambetsariev, Bolot Mambetsariev, Xiwei Wu, Aliya Husain, Everett E. Vokes, Deric L. Wheeler, Ravi Salgia

**Affiliations:** ^1^ Department of Medical Oncology and Therapeutics Research, City of Hope National Medical Center, Duarte, CA, USA; ^2^ Department of Molecular and Cellular Biology, City of Hope National Medical Center, Duarte, CA, USA; ^3^ Department of Medicine, University of Chicago, Chicago, IL, USA; ^4^ Department of Pathology and Laboratory Medicine, University of Wisconsin-Madison, Madison, WI, USA

**Keywords:** exosome, miRNA, NSCLC, SCLC, liquid biopsy

## Abstract

Lung cancer is a devastating disease with overall bleak prognosis. Current methods to diagnose lung cancer are rather invasive and are inadequate to detect the disease at an early stage when treatment is likely to be most effective. In this study, a shotgun sequencing approach was used to study the microRNA (miRNA) cargo of serum-derived exosomes of small cell lung cancer (SCLC) (n=9) and non-small cell lung cancer (NSCLC) (n=11) patients, and healthy controls (n=10). The study has identified 17 miRNA species that are differentially expressed in cancer patients and control subjects. Furthermore, within the patient groups, a set of miRNAs were differentially expressed in exosomal samples obtained before and after chemotherapy treatment. This manuscript demonstrates the potential of exosomal miRNAs for developing noninvasive tests for disease differentiation and treatment monitoring in lung cancer patients.

## INTRODUCTION

Liquid biopsy, or the test of blood samples to look for biological material shed from tumors, is being used increasingly for cancer diagnostics, treatment planning, and monitoring treatment response. Among the entities targeted by liquid biopsy are tumor cells, cell-free DNA and extracellular vesicles, specifically exosomes [1, 2]. Exosomes contain lipids, proteins, DNA, as well as coding and noncoding RNAs [3–5], and reflect the status of the cells from which they originate as well as the cellular mechanisms they engage in [1, 3]. MicroRNAs (miRNAs) are a fraction of short noncoding RNA molecules sized between 19 and 22 base pairs. These small transcripts are capable of modulating various cellular processes and have also been implicated in several pathological phenotypes [6]. miRNAs target the 3′ untranslated regions of messenger RNAs and regulate gene expression both at the translational and post-translational level [7]. In cancer cells, miRNAs function both as tumor suppressors as well as oncogenes [8, 9]. Therefore, it appears quite plausible that exosomal miRNAs may constitute a gene signature that could potentially reveal information about the disease pathobiology and prognosis [10]. Indeed, utilizing miRNAs as biomarkers for early detection and diagnosis have led to a more favorable treatment outcome [11].

Lung cancer is a devastating disease with 228,190 newly diagnosed cases and 159,480 cancer-related deaths in the US in 2016 [12]. Lung cancer is classified into two major groups: small cell lung cancer (SCLC) and non-small cell lung cancer (NSCLC) that accounts for 85% of all lung cancers. In recent years, our understanding of lung cancer has improved significantly. However, the prognosis remains bleak with an overall 5-year survival of only 17% [12] and 7% [13, 14] for NSCLC and SCLC, respectively. Current methods to diagnose lung cancer involve rather invasive procedures [15]. To collect a sufficient sample from a detected lesion, a traditional needle biopsy, bronchoscopy, thoracentesis, or other invasive methods are required depending on the disease site. An accurate diagnosis is more likely when the obtained tissue is adequate for histopathological analysis. However, occurrences of inadequate sample collection make for an unreliable diagnosis, furthering patient risk and discomfort for repeated tissue collection. Therefore, gathering diagnostic information using noninvasive procedures can lessen the reliance on traditional biopsies, easing patients from additional distress.

Recently established methods for plasma DNA analysis have advanced targeted therapies for early-stage NSCLC by detecting biomarkers indicative of actionable mutations, such as EGFR and ALK mutations [16]. Liquid biopsies have also advanced the characterization of additional genetic alterations, such as BRAF, MET exon 14 skipping mutations, and ROS-rearrangements, that suggest effective alternatives for cancer treatment with targeted therapeutics as opposed to traditional cytotoxic chemotherapy [17]. To supplement the genetic biomarker information provided by circulating tumor DNA, exosomal content of miRNA can provide valuable diagnostic information, as seen in prostate cancer [18] and malignant mesothelioma [19]. A simple blood draw and isolation of exosomes allow for the profiling of a patient's miRNA expression signature. This recent approach of analyzing a patient's miRNA profile has the potential to provide insight with regard to lung cancer type, prognosis as well as treatment efficacy.

## RESULTS

**Figure 1:**
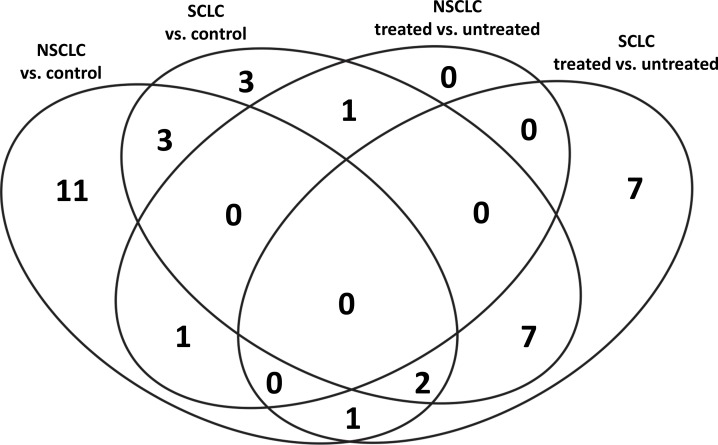
Distribution of miRNA species amongst study groups

The number of sequences successfully aligned to the human genome and the number of mature miRNA detected in different disease and treatment groups are presented in Table 1. Overall, the number of sequences aligned to the human genome varied from 4.7 to 18.4 million with median value 9.4 million per sample. The median percent of sequences aligned to the mature miRNA in SCLC and NSCLC before treatment was 27.9% and 24.8%; SCLC and NSCLC, after treatment, 27.9% and 17.8%; and in the healthy control group, 39.5%. Of these, 36 miRNAs were observed to be differentially expressed between study groups. The Venn diagram (Figure 1) demonstrates the distribution of the miRNA species between disease and treatment groups. Of these, the largest number of miRNA species (n=11) uniquely characterized differences between NSCLC and healthy control subjects. The effect of treatment in the SCLC group was characterized by n=7 unique miRNA species. SCLC subjects were specifically characterized by n=3 miRNA. Interestingly, no miRNAs were unique for NSCLC samples after chemotherapy treatment. Only five miRNAs, or 31% and 27%, were shared between SCLC and NSCLC datasets, suggesting that miRNA content is disease-specific.

**Table 1:** Number of sequences aligned to human genome and to mature miRNA genes in different disease treatment groups

Diagnosis	Treatment	Sequences aligned to human genome (median ± std. error)	Mature miRNA (median ± std. error)
NSCLC	Untreated	7897208 ± 893462.4	632902 ± 124703
NSCLC	Treated	1.22 x10^7^ ± 531804.1	758692 ± 255011
SCLC	Untreated	6249009 ± 1075251	193518 ± 78980.59
SCLC	Treated	7814297 ± 675651	771620 ± 175591
Healthy Control	Untreated	1.01x 10^7^ ± 951995	227399.5 ± 43119

**Figure 2:**
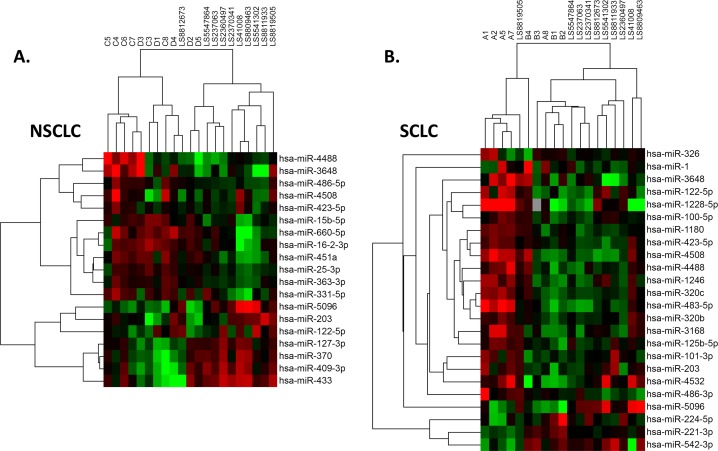
Distribution of case samples before and after chemotherapy, contrasted by samples from healthy control subjects NSCLC **(A)** and SCLC **(B)**.

Unsupervised hierarchical clustering was used to analyze the differentially expressed exosomal miRNA. As shown in Figure 2A, NSCLC and healthy control samples formed four major clusters: the first cluster consisted mostly of untreated NSCLC samples, the second and third clusters were a mixture of treated NSCLC and control samples, and finally, the fourth cluster was made up of only control samples. Figure 2B depicts the distribution of SCLC and control samples and their clustering into three groups: the first cluster contained mostly SCLC untreated samples while the second and third were formed by the treated SCLC and control samples, respectively. Therefore, the content of exosomal miRNA can not only accurately distinguish SCLC and NSCLC patients, but also aid in monitoring the progress treatment.

### Differentiation of samples using exosomal miRNA

The differentially expressed miRNAs were examined to determine their capacity to differentiate case and control samples or to mark therapy progress. ROC analysis was conducted for 18 miRNAs differentially present between NSCLC and control groups, 16 miRNAs that distinguish SCLC and control, and 2 and 17 miRNAs, respectively, that can discern NSCLC and SCLC groups before and after treatment. A separate test was conducted to examine 24 miRNAs differentially expressed between non-treated SCLC and NSCLC specimens. Features with an area under curve >0.8, signifying good and excellent accuracy of prediction, were selected and are presented in Table 2. These analyses revealed 7 miRNAs with “good” and “excellent” prediction accuracy, that can distinguish NSCLC and control samples (hsa-miR-451a, hsa-miR-486-5p, hsa-miR-363-3p, hsa-miR-660-5p, hsa-miR-15b-5p, hsa-miR-25-3p, hsa-miR-16-2-3p); 1 miRNA separating SCLC and control (hsa-miR-1180), and 3 miRNAs separating SCLC samples before and after treatment (hsa-miR-221-3p, hsa-miR-224-5p, hsa-miR-125b-5p). For NSCLC, no miRNAs were found to separate case, control and treated patients. The comparison between SCLC and NSCLC specimens collected before treatment initiation revealed 13 miRNAs (Table 2) capable to correctly distinguish SCLC and NSCLC patients. Of these, 3 miRNAs (hsa-miR-331-5p, hsa-miR-451a, hsa-miR-363-3p) should be noted for an exceptional performance. These 3 miRNAs were able to discriminate SCLC and NSCLC cases with 100% sensitivity, 100% specificity underscoring the potential of miRNAs to serve as good candidates for differentiating NSCLC and SCLC; NSCLC, in contrast to SCLC, appeared to be relatively easy to identify using exosomal miRNA; however it is difficult to follow the progress of treatment using the same approach. The provided data warrant future investigation implementing large discovery and verification cohorts.

**Table 2:** ROC analysis of exosomal miRNA in liquid biopsies

Groups Compared	miRNA	Cut Point	Sensitivity	Specificity	Correctly Classified	AUC
SCLC vs. Control	hsa-miR-1180	( ≥.0000.. )	80.00%	90.00%	86.67%	0.94
	hsa-miR-451a	( ≥.0047.. )	83.33%	100.00%	93.75%	0.98
	hsa-miR-486-5p	( ≥.0038.. )	100.00%	90.00%	93.75%	0.98
	hsa-miR-363-3p	( ≥.000089 )	83.33%	100.00%	93.75%	0.95
NSCLC vs. Control	hsa-miR-660-5p	( ≥ 8.87e.. )	83.33%	100.00%	93.75%	0.91
	hsa-miR-15b-5p	( ≥.000015 )	83.33%	90.00%	87.50%	0.91
	hsa-miR-25-3p	( ≥.000109 )	83.33%	90.00%	87.50%	0.91
	hsa-miR-16-2-3p	( ≥ 6.05e.. )	83.33%	100.00%	93.75%	0.88
	hsa-miR-221-3p	( ≥.0014.. )	100.00%	80.00%	88.89%	0.95
SCLC Treated vs. Untreated	hsa-miR-224-5p	( ≥ 6.86e.. )	100.00%	80.00%	88.89%	0.9
	hsa-miR-125b-5p	( ≥.000045 )	60.00%	100.00%	77.78%	0.8
	hsa-miR-1228-5p	( ≥ 3.56e.. )	80.00%	100.00%	90.91%	0.9333
	hsa-miR-1246	( ≥.0008.. )	60.00%	100.00%	81.82%	0.8333
	hsa-miR-203	( ≥.000022 )	80.00%	100.00%	90.91%	0.8333
	hsa-miR-483-5p	( ≥.0000.. )	80.00%	100.00%	90.91%	0.8333
	hsa-miR-542-3p	( ≥ 3.82e.. )	83.33%	80.00%	81.82%	0.8
	hsa-miR-331-5p	( ≥ 4.67e.. )	100.00%	100.00%	100.00%	1
SCLC vs. NSCLC	hsa-miR-451a	( ≥.0027.. )	100.00%	100.00%	100.00%	1
	hsa-miR-486-5p	( ≥.0076.. )	83.33%	100.00%	90.91%	0.9667
	hsa-miR-660-5p	( ≥ 8.87e.. )	83.33%	100.00%	90.91%	0.8667
	hsa-miR-15b-5p	( ≥.0000.. )	83.33%	100.00%	90.91%	0.9333
	hsa-miR-16-2-3p	( ≥.0000.. )	66.67%	100.00%	81.82%	0.8333
	hsa-miR-25-3p	( ≥.0002.. )	66.67%	100.00%	81.82%	0.8667
	hsa-miR-363-3p	( ≥.0000.. )	100.00%	100.00%	100.00%	1

^*^ miRNA with area under curve (AUC) >0.80 were selected.

### Exosomal miRNA patterns and biological processes in the tumor

Based on the differences observed in exosomal miRNA content between NSCLC and SCLC patients, we hypothesized that the exosomal miRNA could be indicative of the biological differences existing within different types of lung cancer. To gain new insight into the disease biology, we employed the Ingenuity Pathway Analysis (IPA) platform (Table 3). In both cases, the analysis agreed with the top three Diseases and Bio Functions affected in both NSCLC and SCLC groups: Cancer, Organismal Injury and Abnormalities, and Tumor Morphology. However, the fourth and fifth places were different: Respiratory Disease, Reproductive System Disease were seen in NSCLC, and Renal and Urological Disease, Respiratory Disease were seen in SCLC. The top five Molecular and Cellular Functions signified by miRNA in NSCLC were Cell Cycle, DNA Replication, Recombination and Repair, Cell Death and Survival, Cellular Development, and Cellular Growth and Proliferation. For SCLC samples, the top functions were Cellular Assembly and Organization, Cellular Function and Maintenance, Cellular Movement, Cell Morphology, and Cellular Development. The top five canonical pathways in NSCLC were PTEN Signaling, PI3K/AKT Signaling, Molecular Mechanisms of Cancer, Glioblastoma Multiforme Signaling, and Pancreatic Adenocarcinoma Signaling; in SCLC, the top five canonical pathways were Calcium Signaling, Purine Ribonuclease Degradation to Ribose-1-Phosphate, UDP-N-acetyl-D-galactosamine biosynthesis II, Regulation of the Epithelial-Mesenchymal Transition Pathways, and Xanthine and Xanthosine Salvage.

**Table 3:** Biological processes identified by exosomal miRNA

NSCLC				SCLC						SCLC treated				
Diseases and Biological function	p-values			Diseases and Biological Function			p-values			Diseases and Biological Function			p-values	
Cancer	1.1E-7 - 2.8E-20			Cancer			1.4E-3 - 1.17E-8			Organismal Injury and Abnormalities			9.66E-09 - 1.64E-28	
Organismal Injury and Abnormalities	1.1E-7 - 1.33E-19			Organismal Injury and Abnormalities			1.4E-3 - 1.17E-8			Cancer			9.66E-09 - 1.64E-28	
Tumor Morphology	1.43E-8 - 4.51E-18			Tumor Morphology			1.4E-3 - 1.17E-8			Inflammatory Response			1.02E-08 - 1.10E-25	
Respiratory Disease	9.37E-8 5.79E-14			Renal and Urological Disease			9.69E-4 - 2.4E-6			Respiratory Disease			5.92E-09 - 2.44E-23	
Reproductive System Diseae	6.03E-8 - 3.13E-13			Respiratory Disease			1.4E-3 - 3.17E-6			Gastrointestinal Disease			8.29E-09 - 6.12E-22	

Molecular and Cellular Functions			p-values			Molecular and Cellular Functions		p-values			Molecular and Cellular Functions		p-values	
Cell Cycle			7.79E-8 - 7.44E-25			Cellular Assembly and Organization		1.44E-3 - 1.7E-12			Cellular Growth and Proliferation		9.66E-09 - 6.99E-33	
DNA Replication, Recombination, Repair			4.55E-8 - 2.13E-19			Cellular Function and Maintenance		1.44E-3 - 1.7E-12			Cellular Development		9.66E-09 - 5.48E-31	
Cell Death and Survival			1.11E-7 - 2.14E-19			Cellular Movement		1.29E-3 - 1.34E-11			Cell Death and Survival		1.00E-08 - 5.61E-30	
Cellular Development			1.10E-7 - 4.51E-18			Cell Morphology		9.56E-4 - 5.35E-10			Cell Cycle		9.82E-09 - 2.89E-24	
Cellular Growth and Proliferation			9.62E-8 - 4.51E-18			Cellular Development		1.40E-3 - 1.41E-8			Cellular Movement		2.11E-09 - 1.80E-18	

Canonical Pathways		p-values			Canonical Pathways				p-values			Canonical Pathways		p-values
PTEN Signaling		1.77E-14			Calcium Signaling				1.44E-03			Molecular Mechanisms of Cancer		1.04E-10
PI3K/AKT Signaling		3.57E-14			Purine Ribonucleoside Degradation to Ribose-1-phosphate				2.33E-03			Glioblastoma Multiforme Signaling		2.22E-10
Molecular Mechanisms of Cancer		6.51E-13			UDP=N-acetyl-D-galactosamine Biosynthesis II				4.50E-03			Glioblastoma Signaling		2.15E-08
Glioblastoma Multiforme Signaling		2.26E-12			Regulation of the Epithelial-Mesenchymal Transition Pathway				8.73E-03			PI3K/AKT Signaling		6.11E-08
Pancreatic Adenocarcinoma Signaling		3.66E-12			Xanthine and Xanthosine Salvage				9.33E-03			Glucocorticoid Receptor Signaling		1.68E-07

A similar analysis applied to the set of SCLC exosomal miRNAs affected by treatment revealed biological alterations associated with the therapeutic intervention. The changes in the list of top five Diseases and Disorders categories included a switch of “Organismal Injury and Abnormalities” and “Cancer” between the first and second place and appearance of Inflammatory Response within the list of top five categories. On the other hand, the list describing Molecular and Cellular Functions underscored the strong effect of treatment; the top categories affected by treatment were Cellular Growth and Proliferation, Cellular Development, Cell Death and Survival, Cell Cycle, Cellular Movement. The top Canonical Pathways affected by SCLC treatment were Molecular Mechanisms of Cancer, Glioblastoma Multiforme Signaling, Glioblastoma Signaling, PI3K/AKT Signaling, and Glucocorticoid Receptor Signaling. In summary, the analysis suggests that the treatment affects the functional profile of tumor and causes the rise of inflammatory processes.

### IPA molecular activity prediction modeling and model verification

**Figure 3:**
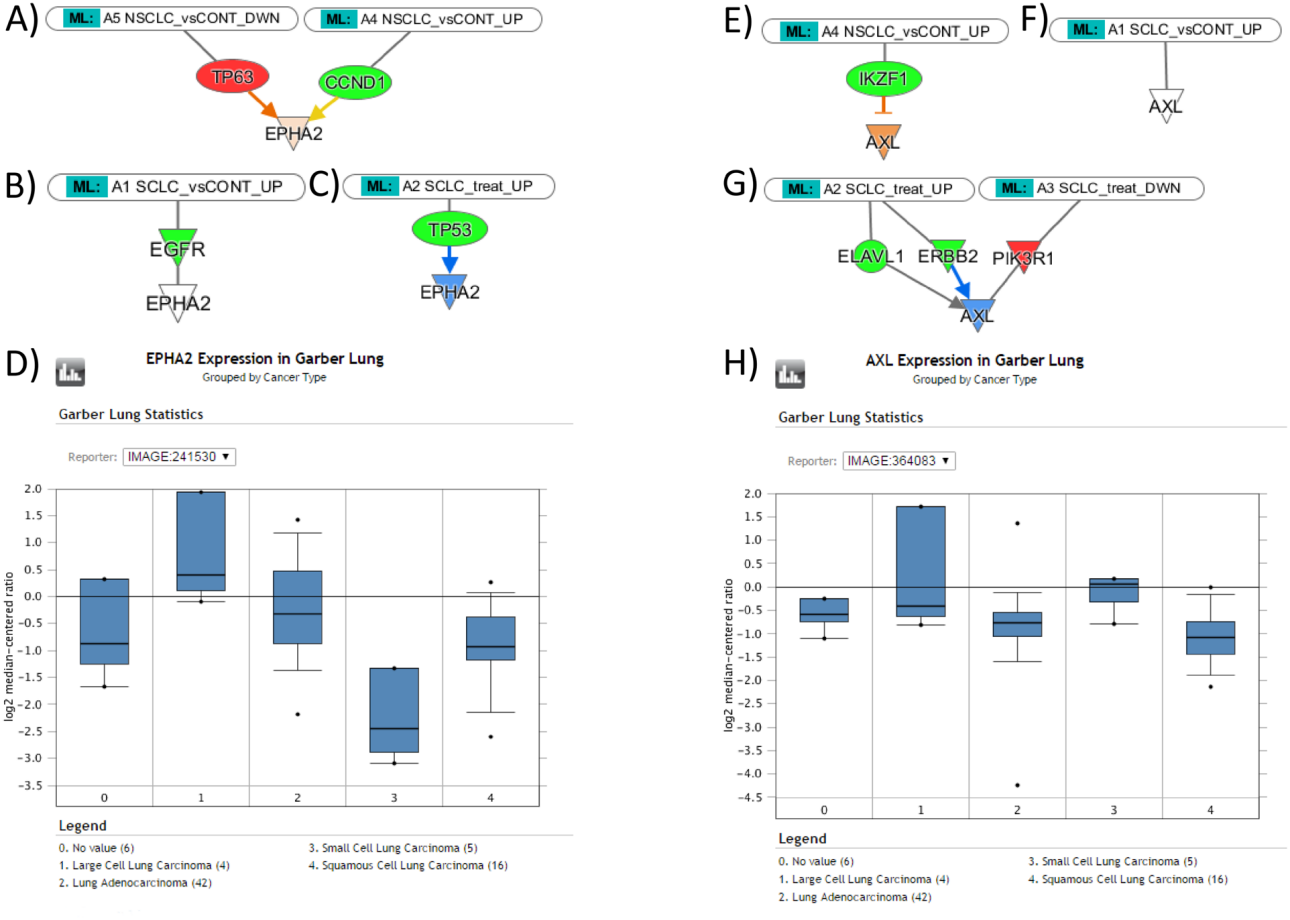
The expression levels of EPHA2 and AXL genes in NSCLC and SCLC tissues, as suggested by Molecular Activity Prediction algorithm (IPA) in response to miRNA influence, and the data of gene expression profiling from Oncomine **(A)** Predicted upregulation of EPHA2 gene in NSCLC tissue; **(B)** the lower level of expression of EPHA2 gene in SCLC; **(C)** Predicted downregulation of EPHA2 gene in response to treatment; **(D)** Oncomine reveals higher levels of EPHA2 gene expression in lung adenocarcinoma and squamous cell carcinoma than in small cell lung carcinoma, as consistent with predicted patterns. **(E)** Predicted upregulation of AXL gene in NSCLC; **(F)** the lower level of EPHA2 gene in SCLC; **(G)** Predicted downregulation of AXL gene in response to treatment; **(H)** Oncomine reveals higher levels of AXL gene expression in small cell lung carcinoma than in lung adenocarcinoma and squamous cell carcinoma, contradictory to the predicted patterns.

The hypothesis that the exosomal miRNA cargo reflects the patterns of gene expression in tumor tissue was tested further using the Molecular Activity Prediction algorithm implemented in IPA software. The assumption was made that elevated miRNA leads to suppression of target genes and that decrease of miRNA facilitates expression of target genes. The activity prediction algorithm was then implemented for the regulatory networks of six important lung cancer oncogenes (FAK, PXN, MET, RON (MST1R), EPHA2, AXL). Of them, focal adhesion kinase (FAK) is a non-receptor tyrosine kinase, upregulated in NSCLCs, and involved in neoplastic transformation, invasion, and metastases, such as cell adhesion, migration and apoptosis [20]; PXN is known to be associated with lung adenocarcinoma progression [21]; MET is important in promoting tumor growth, progression and invasion in lung cancers [22]; RON (MST1R) is involved in tumor growth and metastasis [23, 24]; EPHA2 is overexpressed in 70% of NSCLC and strongly associated with patient's survival [25]; AXL is an emerging drug target in NSCLC and SCLC [26, 27]. Figure 3 exemplified the results of this analysis for EPHA2 and AXL genes.

In summary (Table 4), the prediction suggests that EPHA2 is upregulated in NSCLC through the repression of TP63 and suppression of CCND1; in SCLC, the suppression of EGFR does not affect EPHA2; therefore, we should expect higher level of EPHA2 in NSCLC samples. During the treatment of SCLC, EPHA2 is suppressed via downregulation of TP53. FAK in NSCLC is suppressed via EGFR, and downregulation of IGF1R and integrin; in SCLC, suppression is achieved via downregulation of MET and EGFR; therefore, the model predicts that FAK expression has no difference in NSCLC and SCLC. During the SCLC treatment, FAK is suppressed via downregulation of ERBB2, IGF1R, and MYCN. PXN in NSCLC is suppressed via ITGA5 and IGF1R downregulation, stimulatory effects of ABL1 upregulation does not affect resulting trend towards the gene suppression; in SCLC, suppression is achieved via downregulation of NRP1; therefore we should expect a higher level of PXN in NSCLC than in SCLC specimens. During the treatment of SCLC, PXN is suppressed via downregulation of IGF1R, CCR5, and ABL1. MET in NSCLC is suppressed via EGFR, JUN, F2 downregulation, stimulatory effects of P38MAPK upregulation does not affect resulting trend towards the MET suppression; in SCLC, suppression is achieved via combined downregulation of F2, EGFR, ITGB4, NRP1, P38MAPK; therefore, we should expect a higher level of MET in NSCLC than in SCLC specimens. The treatment of SCLC upregulates PXN expression via upregulation of FOS and downregulation of P38MAPK. RON (MST1R) in NSCLC was unaffected, while in SCLC, RON upregulation was achieved via ESR1 suppression; therefore, we should expect a higher level of RON in SCLC specimens. The treatment of SCLC suppressed RON via upregulation of ESR1. AXL in NSCLC, was upregulated through the IKZF1 suppression, while in SCLC it was unaffected; therefore, we should expect a higher level of AXL in NSCLC specimens. The treatment of SCLC suppressed AXL via downregulation of ERBB2.

**Table 4:** The results of Molecular Activity Prediction algorithm (IPA) predicting the effect of miRNA on the expression levels of genes in NSCLC and SCLC tissues

Group	Effector Molecules	Predicted Effect
NSCLC	TP63 ↑ CCND1 ↓	EPHA2 ↑
	EGFR ↓ IGF1R ↓ Integrin ↓	FAK ↓
	ITGA5 ↓ IGF1R ↓ ABL1 ↑	PXN ↓
	EGFR ↓ JUN ↓ F2 ↓ P38MAPK ↑	MET ↓
		RON (MST1R) (.)
SCLC	IKZF1 ↓	AXL ↑
	EGFR ↓	EPHA2 (.)
	MET ↓ EGFR ↓	FAK ↓
	NRP1 ↓	PXN ↓
	F2 ↓ EGFR ↓ ITGB4 ↓ NRP1 ↓ P38MAPK ↓	MET ↓
	ESR1 ↓	RON (MST1R) ↑
		AXL (.)
SCLC Treated	TP53 ↓	EPHA2 ↓
	ERBB2 ↓ IGF1R ↓ MYCN ↓	FAK ↓
	IGF1R ↓ CCR5 ↓ ABL1 ↓	PXN ↓
	FOS ↑ P38MAPK ↓	MET ↑
	ESR1 ↑	RON (MST1R) ↓
	ERBB2 ↓	AXL ↓

↑- upregulation, ↓-downregulation, (.) - no effect.

The prediction was verified in Oncomine using the relevant NCBI GEO dataset GSE3398; gene expression was compared between Lung Adenocarcinoma and Small Cell Lung Carcinoma cases. According to the algorithm, the levels of AXL, EPHA2, MET and PXN expression were greater in NSCLC, the expression of RON was greater in SCLC, and the model predicted no difference in levels of FAK expression between NSCLC and SCLC. Consistent with the prediction, with the exception of AXL and RON, gene expression data from Oncomine confirmed the expression patterns for EPHA2, MET, PXN, and FAK, indicating that the model delivers accurate prediction for 2/3 of the genes.

### Biological function of individual exosomal miRNA

To understand biological functions, miRNAs exhibiting differential expression above 2 logs fold of magnitude were selected. The development of NSCLC was associated with an increase of molecules associated with endoplasmic reticulum (ER) stress [28] - hsa-miR-3648 (up 2.08 folds) and TNF-α, IL-6 suppression [29] - hsa-miR-4488 (up 3.49 folds), and decrease of one that is known to regulate glioma cell invasiveness and the release of extracellular vesicles - hsa-miR-5096 (down 3.02 folds) [30–32].

In SCLC, several molecules were elevated: the marker of breast cancer chemotherapy resistance and self-renewal capability - hsa-miR-4508 (up 2.07 folds) [33], serum-based biomarker for muscle-invasive bladder cancer survival [34] - hsa-miR-486-3p (up 2.16 folds), the noted above as an indicator of the ER stress [28] and a suppressor of antitumor adenomatous polyposis coli 2 (APC2) [28] - hsa-miR-3648 (up 2.2 folds), a critical β-catenin-activated prometastatic miRNA and a negative regulator of the metastasis suppressors RhoGDI1 and ALCAM [35] - hsa-miR-483-5p (up 3.16 folds), apoptosis suppressing [36] - hsa-miR-1228-5p (up 3.29 folds). The hsa-miR-5096 (down 2.76 folds) of unknown function was downregulated.

In the case of SCLC, treatment lead to elevation of hsa-miR-1228-5p (up 5.17 folds), hsa-miR-483-5p (up 3.45 folds), and the implicated in breast cancer chemotherapy resistance [33] - hsa-miR-4532 (up 2.4 folds), as well as hsa-miR-3168 (up 2.4 folds) of unknown function. In contrast, the level of tumor suppressive hsa-miR-542-3p (down 2.04 folds) [37–40] was downregulated.

The treatment of NSCLC was not associated with any significant changes in exosomal miRNA content. Two of the most affected miRNA molecules, hsa-miR-423-5p, known as a positive regulator of autophagy in hepatocellular carcinoma [41] and the regulator of cell proliferation of gastric cancer cells [42], and hsa-miR-331-5p, connected to chemotherapy resistance and relapse in leukemia [43], were upregulated but only by 1.06 and 1.18 logs fold magnitude, respectively.

## DISCUSSION

The cargo of exosomes reflects biological processes taking place inside the cells of origin [3] and the status of the important oncogenes such as EGFR and KRAS [44]. It is therefore not surprising that the cancer related functional categories were among the top of the list (Table 3), which is consistent with the relatively high success rate of our molecular function modeling. Similarly, the neuroendocrine origin of SCLC may help explain the abundance of glioblastoma-related canonical pathways affected by SCLC treatment. The fact that the Akt/PKB signaling pathway [45] was affected by SCLC treatment suggests the presence of ongoing an apoptotic process.

The disagreement between predicted and observed behavior of several genes deserves a special comment. Recent studies demonstrate overexpression of the receptor tyrosine kinase AXL in lung adenocarcinoma tumor tissues compared with adjacent lung tissues [27] and strong association of AXL expression with tumor invasiveness [46]. Therefore, the predicted upregulation of AXL in NSCLC samples matches well with the provided literature data, but contradicts with the data from the Oncomine dataset. Moreover, the available literature suggest that in SCLC the level of intrinsic AXL expression is low [47] which aligns well with the predicted effects of exosomal miRNA and again contradicts the data from Oncomine. Potential explanation of this discrepancy could come from the available data suggesting that two patient populations, one with a low and one with a high level of AXL [26], exist within the SCLC cohort. The information about MST1R expression in SCLC and NSCLC is similarly perplexing. The publication by Cervantes et al. [48] describes MST1R expression in lung tumors and demonstrates the elevated levels of MST1R expression in lung tumors of neuroendocrine origin (SCLC). This observation contradicts Oncomine data where the higher levels of MST1R were found for NSCLC but not for SCLC. The provided examples suggest that the success rate of *in silico* modeling could be higher if verified against matching biopsy tissues rather than a contradictory Oncomine reference set.

The utility of miRNA signatures for lung cancer diagnosis and prognosis is well established [49–53]. Therefore, use of miRNAs has increased markedly over the past several years especially due to the non-invasive nature of circulating miRNA analysis in sera [54, 55] or sputum [56]. Recently, a study reported that about half of lung cancer diagnoses is detected at a late stage of the disease (III/IV) [12]. Thus, miRNAs have the potential to fulfill a critical need for early detection. For example, elevated plasma levels of miR-21, miR-126, miR-210, and miR-486 were reported in association with stage I lung cancer [57]. In addition, miRNA signatures may also serve as a practical diagnostic approach for identifying various NSCLC subtypes; high levels of miR-205 were seen in patients with squamous cell carcinoma distinguishing these patients from those with other subtypes of NSCLC [58, 59].

miRNA profiles have also been utilized as a prognostic biomarker for lung cancer progression. For instance, a group of 34 miRNAs detected in the serum can determine asymptomatic individuals with early-stage lung cancer at risk for progression to an advanced stage [60]. Furthermore, the expression patterns of several miRNAs (let-7, miR-221, miR-137, miR-372, and miR-182) show a positive correlation with survival rates in lung cancer patients [53, 61]. Similarly, another study indicated that the expression levels of a set of 11 miRNAs that included miR-486, miR-30d, miR-1, and miR-499, were significantly correlated with disease prognosis [62]. In adenocarcinoma patients, a group of 32 miRNAs was highly expressed in tumor tissue, and more specifically, let-73, miR-25, miR-191, miR-34a, and miR-34c were correlated with prognosis [63]. Another practical miRNA signature of miR-21 and miR-24 pre- and post-operation [64] showed promise as potential biomarkers for cancer recurrence.

In contrast to NSCLC, there are currently very few studies that have identified miRNA specific for SCLC using peripheral blood. For example, Nishikawa et al. reported elevated miR-375 [65] in lung neuroendocrine carcinoma, Yu et al. suggested miR-92a-2 [66] as a SCLC biomarker, and Demes et al. [67] proposed 2 miRNAs (miR-21 and miR-34a) to differentiate various types of neuroendocrine tumors in lung. Thus, to the best of our knowledge, the present study represents a significant advancement in identifying miRNAs that can help discern SCLC and NSCLC with high specificity and sensitivity.

Of note, none of the molecules seen in the present study featured as lung cancer biomarkers in the large report of Inamura [50]. Of the 18 miRNA described in our study, only 3 were previously reported in regard to lung cancer diagnostics. For example, Xiance et al. [49] found hsa-miR-486-5p upregulated, and hsa-miR-15b-5p to be downregulated in blood-derived exosomes from adenocarcinoma and squamous cell carcinoma patients, and Rabinowits et al. [68] found hsa-miR-203 in blood-derived exosomes and reported it as biomarker for lung adenocarcinoma. Notably, of the three molecules found in the blood circulating exosomes, two (hsa-miR-486-5p, hsa-miR-15b-5p) are in agreement with our findings in NSCLC. In our study, hsa-miR-203 was not specific enough to differentiate NSCLC from healthy subjects but was capable of discriminating NSCLC and SCLC cases (sensitivity 80%, specificity 100%) with the same effectiveness as hsa-miR-486-5p (sensitivity 83%, specificity 100%). One of the possible explanations of this phenomenon is a difference in miRNA content of exosomes and miRNA derived from tissues and serum. The study by Zhao et al., (2016) demonstrated higher complexity of miRNA profiles in bovine sera than in sera-derived exosomes [69] with several miRNA species exclusive for each compartment. Similar results were demonstrated by Lim et al., (2017) [70] who demonstrated difference in miRNA profiles between cells and exosomes harvested from cell medium. Hence, in the future, we could see a higher number of matches between datasets once additional lung cancer-related studies that are based on blood-derived exosomes become available.

In this study, the miRNA species from blood-derived exosomes were studied in lung cancer patients (NSCLC and SCLC) and a group of healthy control individuals. We have demonstrated that exosomal cargo is different between patients with different types of cancer as well as between tumor-bearing and control individuals. We have demonstrated the change in exosomal miRNA profiles of patients who underwent chemotherapy treatment—the effect was stronger in the SCLC cohort. Our analysis suggests that exosomal miRNA could serve as a potential marker of biological processes within a tumor, can differentiate patients, and mark chemotherapy response in lung cancer patients.

## MATERIALS AND METHODS

### Study cohort

The study cohort comprised 30 subjects (Table 5) that included 11 NSCLC patients (untreated n=6, 5 male, 1 female, median age 76 years; treated n=5, 2 male, 3 female, median age 65 years), 9 SCLC patients (untreated n=5, 4 male, 1 female, median age 72; treated n=4, 3 male, 1 female, median age 65.5) and 10 healthy control subjects (5 male, 5 female, median age 27 years). Serum samples from the patients were collected in the University of Chicago (Chicago, IL). Healthy donor sera were purchased from Innovative Research Inc. (Novi, MI). Study was conducted under IRB 9571 and 13473. All patient subjects provided informed consent.

**Table 5:** Study group demographics

Group	Sample ID	Histology	Age (years)	Sex	Chemotherapy Treatment
Untreated NSCLC	C3	Adenocarcinoma	77	F	
	C4	Adenocarcinoma	83	M	
	C5	Adenocarcinoma	75	M	
	C6	Adenocarcinoma	65	M	
	C7	Adenocarcinoma	58	M	
	C8	Adenocarcinoma	78	M	
Treated NSCLC	D1	Adenocarcinoma	69	M	Carboplatin, Taxotere, Pemetrexed
	D2	Adenocarcinoma	65	F	Bevacizumab, Cisplatin, Navelbine, Carboplatin, Taxotere, Pemetrexed
	D3	Adenocarcinoma	77	M	Cisplatin, 5-Fluorouracil
	D4	Adenocarcinoma	63	F	Carboplatin, Pemetrexed
	D5	Adenocarcinoma	60	F	Cisplatin, Pemetrexed
Untreated SCLC	A1	Small cell carcinoma	68	M	
	A2	Small cell carcinoma	80	M	
	A5	Small cell carcinoma	72	M	
	A7	Small cell carcinoma	70	F	
	A8	Small cell carcinoma	81	M	
Treated SCLC	B1	Small cell carcinoma	62	F	Carboplatin, Etoposide
	B2	Small cell carcinoma	70	M	Carboplatin, Etoposide, Sunitinib
	B3	Small cell carcinoma	61	M	Cisplatin, Etoposide, Carboplatin, Topotecan
	B4	Small cell carcinoma	69	M	Cisplatin, Vinorelbine
Healthy Control	LS8812673		24	F	
	LS5547864		27	F	
	LS5541008		26	F	
	LS2370663		27	F	
	LS2370341		25	F	
	LS2360497		29	M	
	LS5541302		28	M	
	LS8809463		26	M	
	LS8811933		28	M	
	LS8819505		38	M	

### miRNA sequencing and statistical analysis

Exosomal RNA was extracted from 500 μl of serum using exoRNeasy Serum/Plasma RNA kit for purification of RNA from exosomes and other extracellular vesicles out of serum or plasma samples (Qiagen, USA) [71, 72] according to manufacturer's instructions. Library preparation, as well as cluster generation and deep sequencing, were performed according to the 5' ligation-dependent (5' monophosphate-dependent) protocol as described by the manufacturer (Digital Gene Expression for small RNA; Illumina, San Diego, CA, USA). For each sample, 5 μl of total exosomal RNA extracted from serum was used for small RNA library preparation. Small RNAs were size-selected between 17 and 52 nt., according to the single-stranded DNA marker in the small RNA sequencing kit (Illumina). The library was quantified using picoGreen and qPCR. Sequencing was performed on a Hiseq 2500 (Illumina). Image processing and base calling were conducted using Illumina's pipeline.

Sequence data analysis and statistical comparisons were carried out using Bioconductor packages and an in-house developed analysis pipeline using R statistical environment. After mapping the deep sequencing data onto the human genome and counting the reads for the mature miRNAs in the miRBase database, raw miRNA expression data were normalized, and differential expression analysis was performed by Bioconductor package “edgeR.” Significant miRNAs were selected when fold change was more than 2 or less than 0.5, and FDR ≤ 0.05. Heatmaps were generated using Cluster v3.0.

Receiver operator characteristic (ROC) analysis was conducted in STATA v12 using miRNA counts standardized to the count of mature miRNA in the sample. The models of gene signaling networks and molecular activity prediction were created using IPA software.
